# Lipome géant de la cuisse avec signes de souffrance nerveuse - à propos d'un cas

**DOI:** 10.11604/pamj.2014.18.296.5194

**Published:** 2014-08-14

**Authors:** Aniss Chagou, Abdelatif Benbouha

**Affiliations:** 1 Service de Chirurgie Orthopédique et Traumatologique, Centre Hospitalier Universitaire Avicenne, Rabat, Maroc

**Keywords:** Lipomes, tumeur, cuisse, Lipoma, tumor, thigh

## Image en medicine

Les lipomes géants sont des tumeurs adipeuses bénignes, d’évolution lente,ils peuvent être volumineux chez l'adulte, et devenir gênants sans pour autant être malins. Ces tumeurs surviennent généralement entre 22 et 75 ans avec une légère prédominance féminine et avec une durée d’évolution variant entre 2 mois et 40 ans. Nous rapportons le cas d'un patient âgé de 35 ans, qui présentait une tuméfaction indolore de la cuisse gauche évoluant depuis 10 ans augmentant progressivement de volume (B). L'augmentation de volume de la tuméfaction s'est accompagnée de l'apparition de signes de souffrance nerveuse sur le territoire du nerf sciatique. Le tout évoluant dans un contexte de conservation de l’état général. A ce stade nous avions trois diagnostics possibles, un lipome qui était le plus probable, un liposarcome ou bien une tumeur nerveuse. L'IRM n'a pas trouvé de signes de malignité, la séquence Fatsata confirmé la nature graisseuse de la tumeur. Nous avons réalisé une biopsie par abord antéro-interne (centrée sur la tumeur) qui a confirmé le résultat de l'IRM. L'excision la tumeur en sa totalité a été réalisée en emportant la cicatrice de la biopsie et en respectant tout les éléments anatomiques de la cuisse qui étaient refoulés. Le lipome pesait 2500 grammes et mesurait 28x16 cm (B).

**Figure 1:**
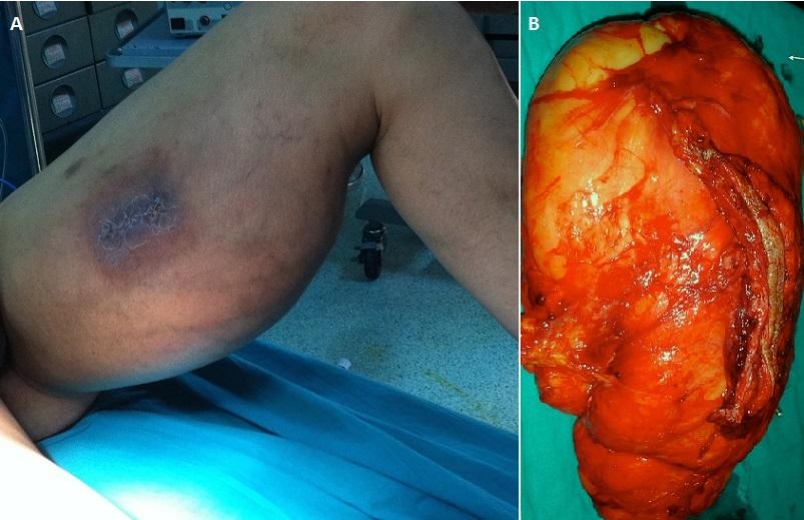
A) tuméfaction indolore de la cuisse gauche avec cicatrice de la biopsie antéro-interne; B) pièce opératoire après excision de la tumeur en sa totalité

